# Neutrophil extracellular traps and neutrophil-related mediators in human thyroid cancer

**DOI:** 10.3389/fimmu.2023.1167404

**Published:** 2023-08-29

**Authors:** Luca Modestino, Leonardo Cristinziano, Remo Poto, Annagioia Ventrici, Marialuisa Trocchia, Silvia Martina Ferrari, Poupak Fallahi, Sabrina Rosaria Paparo, Gianni Marone, Alessandro Antonelli, Gilda Varricchi, Maria Rosaria Galdiero

**Affiliations:** ^1^ Department of Translational Medical Sciences (DiSMeT), University of Naples Federico II, Naples, Italy; ^2^ Center for Basic and Clinical Immunology Research (CISI), University of Naples Federico II, Naples, Italy; ^3^ World Allergy Organization Center of Excellence, University of Naples Federico II, Naples, Italy; ^4^ Department of Clinical and Experimental Medicine, University of Pisa, Pisa, Italy; ^5^ Department of Translational Research and New Technologies in Medicine and Surgery, University of Pisa, Pisa, Italy; ^6^ Department of Surgical, Medical and Molecular Pathology and Critical Area, University of Pisa, Pisa, Italy; ^7^ Institute of Experimental Endocrinology and Oncology (IEOS), National Research Council (CNR), Naples, Italy

**Keywords:** neutrophils, neutrophil extracellular traps, thyroid cancer, dedifferentiated thyroid cancer, NETs biomarkers

## Abstract

**Background:**

Polymorphonuclear neutrophils (PMNs) are the main effector cells in inflammatory responses and play multiple roles in thyroid cancer (TC). PMNs contain and release a plethora of mediators, including granular enzymes [e.g., myeloperoxidase (MPO), pentraxin-3 (PTX3) and matrix metalloproteinase-9 (MMP-9)], and neutrophil extracellular traps (NETs). The aim of this study was to evaluate NETs and neutrophil-derived mediators as possible biomarkers in TC patients.

**Methods:**

20 patients with differentiated thyroid cancer (DTC), 26 patients with dedifferentiated thyroid cancer (De-DTC), 26 patients with multinodular goiter (MNG) and 22 healthy controls (HCs) were recruited. Serum concentrations of free DNA (dsDNA), nucleosomes, citrullinated histone H3 (CitH3) and MPO-DNA complexes were evaluated as NET biomarkers. Neutrophil-related mediators such as MPO, PTX3, MMP-9, CXCL8, and granulocyte-monocyte colony-stimulating factor (GM-CSF) were measured by ELISA.

**Results:**

Serum levels of all four NET biomarkers were increased in DeDTC patients compared to HCs. CitH3 serum levels were selectively increased in both DeDTC and DTC patients compared to HCs and MNG patients. MPO-DNA complexes and nucleosomes were selectively increased only in DeDTC patients compared to HCs and MNG patients. Moreover, MPO-DNA complexes were selectively increased in DeDTC patients compared to DTC patients also. MPO circulating levels were selectively increased in the DeDTC patient subgroup compared to HCs. Circulating levels of PTX3, MMP-9 and GM-CSF were increased in DTC and DeDTC patients compared to HCs. Nucleosomes positively correlated with dsDNA, CitH3, MPO and CXCL8. MPO-DNA complexes positively correlated with dsDNA, CitH3, CXCL8, MPO and nucleosome levels. Moreover, three out of the four NET biomarkers (i.e., dsDNA, nucleosomes and MPO-DNA complexes) were increased in elderly patients compared to young patients and in patients with metastatic disease at diagnosis compared to non metastatic patients. Nucleosomes were higher in males compared to females.

**Conclusion:**

MPO-DNA complexes, nucleosomes and, to some extent, CitH3 levels seem to correlate with malignancy and severity of progressive TC. Moreover, serum concentrations of PMN-related mediators (MPO, PTX3, GM-CSF) were increased in TCs compared to MNG and HCs.

## Introduction

Thyroid cancer (TC) is one of the most common endocrine tumors (1) and its occurrence has risen in the last decade (2). Differentiated thyroid cancer (DTC) arises from thyroid follicular cells and includes papillary thyroid carcinoma (PTC) and follicular thyroid carcinoma (FTC). Anaplastic thyroid cancer (ATC) accounts for 1% of TCs, but is highly aggressive, leading to 15–40% of TC death (3–6). The overall survival (OS) for DTC patients is generally high, but it depends on the cancer subtype and the age of diagnosis. By contrast, the median survival of ATC patients is approximately 6 months (3–6). Almost 50% of ATC occurs after a history of thyroid nodules, PTC or FTC (7). The gold standard therapy for DTC is surgery, while aggressive PTC and FTC need subsequent radioactive iodine (RAI) ablation of the remnants. Following surgery, RAI, and TSH suppressive therapy, the 5-year OS rate of DTC patients is around 98%, even though loco-regional recurrences occur in up to 10–20% of cases, and distant metastases in about 10% (8, 9). Dedifferentiated DTC (DeDTC) patients are PTC or FTC patients, previously treated with surgery and/or RAI, who need a subsequent operation for local recurrence or neck lymph node metastases. 2/3 of patients with DeDTC become RAI-refractory and display a 3-year OS rate lower than 50% (10). Early characterization of thyroid nodules and/or the identification of cellular and humoral factors that predict patients with DTC who will progress to ATC or become RAI-refractory has become a top priority (8).

Chronic low-grade inflammation is linked to tumorigenesis (7, 11) and the relationship between TC and chronic inflammation has begun to be elucidated (12). Inflammatory mediators and immune cells promote TC initiation and progression (13). Several proinflammatory stimuli release a wide spectrum of inflammatory mediators (i.e., CXCL1, CXCL8, CXCL9 and CXCL10) from thyrocytes, which recruit leukocytes (14–18).

Neutrophils (or polymorphonuclear leukocytes – PMNs) play pivotal roles during various phases of the inflammatory response (19, 20) and cancer progression (19, 21). Recent evidence highlights the role of neutrophils in TC. TC-derived soluble factors (TC-conditioned media: TC-CM) promoted human neutrophil chemotaxis through CXCL8 and survival through granulocyte-macrophage colony-stimulating factor (GM-CSF) (22). TC-CM induced activation of neutrophils and modified neutrophils’ kinetic properties. Furthermore, TC-CM induced the production of reactive oxygen species (ROS), expression of proinflammatory and angiogenic mediators (VEGF-A, CXCL8, and TNF-α) and matrix metalloproteinase 9 (MMP-9) (22). Importantly, intratumor neutrophil density correlated with larger tumor size in TC patients (22).

Activated human neutrophils release extracellular fibrillary networks, termed neutrophil extracellular traps (NETs), composed of nuclear elements (DNA and histones) and granule proteins [e.g., myeloperoxidase (MPO), elastase (NE), pentraxin-3 (PTX3)] (23). Although NETs were initially described as playing a protective role against pathogens (24), increasing evidences demonstrate their involvement in cancer (25). NETs promote tumor growth (26, 27), cancer-associated thrombosis (28) and the formation of metastases (29). ATC CM selectively induced NET release, suggesting that NETs could be involved in acquiring a more aggressive behavior by cancer cells (30). Circulating levels of putative NET biomarkers have been evaluated as prognostic biomarkers in several cancers (31–33).

In this study, we evaluated the circulating levels of several NET-derived biomarkers and neutrophil-derived mediators in patients with DTC and De-DTC and healthy controls. NET biomarkers were examined using four techniques (dsDNA, nucleosomes, citrullinated histone H3 and MPO-DNA complexes) with different degrees of specificity.

## Materials and methods

### Patients enrollment

Twenty patients with differentiated thyroid cancer (DTC), 26 patients with dedifferentiated thyroid cancer (DeDTC), 26 patients with multinodular goiter (MNG) and 22 healthy donors, aged >18 years, sex and age matched were recruited at the Department of Clinical and Experimental Medicine of University of Pisa. The ethnic distribution among the groups was comparable, with all participants being white. The clinic-pathological features of the enrolled patients were included in the **Supplementary Table 1**. The study was approved by the Ethics Committee for Clinical trials of Pisa University Hospital and was conducted in accordance with the international standards of good clinical practice and with the provisions of the Declaration of Helsinki. All participants of this study signed the informed consent. We enrolled patients with DeDTC, or DTC undergoing primary thyroidectomy, excision of recurrent tumor, or lymphadenectomy, for recurrent or metastatic TC. Patients recruited met the following inclusion criteria: aged 18-75, diagnosis of DeDTC or DTC histologically confirmed. Exclusion criteria included: infections, other cancers or immune-related diseases, anti-thyroid or immune-suppressive therapy, previous chemotherapy or tyrosine kinase inhibitors. Standard American Joint Committee on Cancer (7th edition) tumor scoring was used for TC staging (34). Pathology reports examined tumor type, size, invasion, and lymph nodes (LN) metastases. Hematoxylin and eosin stains of LN sections evaluated the presence, size, and invasiveness of tumor metastases. Peripheral blood samples were collected at the Department of Clinical and Experimental Medicine of the University of Pisa and were immediately processed. Serum samples were obtained (+ 4°C, 400 g, 20 min), harvested and stored (- 80°C) until used.

### Detection of serum NET biomarkers

Serum levels of free DNA (dsDNA), DNA fragments (mono- and oligonucleosomes) and Citrullinated Histone H3 (CitH3) were measured in TC patients and healthy controls (HCs). Quant-iT™ PicoGreen dsDNA Assay Kit (Thermo Fisher Scientific, Waltham, MA, USA) was used to measure the circulating levels of dsDNA. Enspire Multimode Plate Reader (Perkin Elmer, Waltham, MA, USA) was used to determine dsDNA sample absorbance at 520 nm. Concentrations of mono- and oligonucleosomes were measured using Cell Death Detection ELISA kit (Roche, Basel, Swiss) and CitH3 using ELISA kit (Cayman Chemicals, Ann Arbor, MI, USA). This kit used a specific monoclonal antibody (clone 11D3) against histone H3 citrullinated at residues R2, R8 and R17. A microplate reader (Tecan, Grodig, Austria, GmbH) was used to determine the mono- and oligonucleosomes and CitH3 sample absorbance, respectively at 405 nm and 450 nm. The ELISA sensitivity ranges were 25 pg/ml - 1000 ng/ml (dsDNA) and 0.15 – 10 ng/ml (CitH3). Serum MPO-DNA complex levels were measured as previously described (35). Briefly, 96-well microplates (Thermo Fisher Scientific, Waltham, MA, USA) were coated with the monoclonal mouse anti-human MPO antibody (5 μg/ml; Bio-Rad, CA, USA) diluted in phosphate-buffered saline (PBS) overnight at 4°C. Following blocking with 1% of bovine serum albumin (BSA) in PBS, the samples and the anti-DNA-POD antibody in the Cell Death Detection ELISA kit (Roche, Basel, Swiss) were added to the wells for 2 hours at 22°C, respectively. After incubations, substrate solution from Cell Death Detection ELISA kit (Roche, Basel, Swiss) was added, and the absorbance was measured at 405 nm with a microplate reader (Tecan, Grodig, Austria, GmbH).

### Serum levels of neutrophil-related mediators

Serum concentrations of MPO, MMP-9, PTX3, CXCL8/IL-8 and GM-CSF of patients and HCs were measured using ELISA kit according to the manufacturer’s instructions (DuoSet ELISA kit, R&D System, Minneapolis, MN, USA). A microplate reader (Tecan, Grodig, Austria, GmbH) was used to determine sample absorbance at 450 nm. The ELISA detection ranges were 62.5-4000 pg/ml (MPO), 31.2-2000 pg/ml (MMP-9), 15.6-1000 pg/ml (PTX3), 31.2-2000 pg/ml (CXCL8) and 7.80-500 pg/ml (GM-CSF).

### Statistical analysis

Data were analyzed using GraphPad Prism 8 software package (GraphPad Software, La Jolla, CA, USA). Data are expressed as mean ± SD of the indicated number of patients. Data were tested for normality using D’Agostino & Pearson normality test. If normality was not rejected at 0.05 significance level, we used parametric tests. Otherwise, for not-normally distributed data we used non-parametric tests. Repeated measures one-way analysis of variance (ANOVA) was used where appropriated and described in the figure legends. Correlations between two variables were assessed by Pearson and/or Spearman rank correlation analysis and reported as coefficient of correlation (r). Differences were assumed to be statistically significant when *p* < 0.05.

## Results

### TC patients display increased circulating levels of NET biomarkers compared to HCs

**Figure 1:**
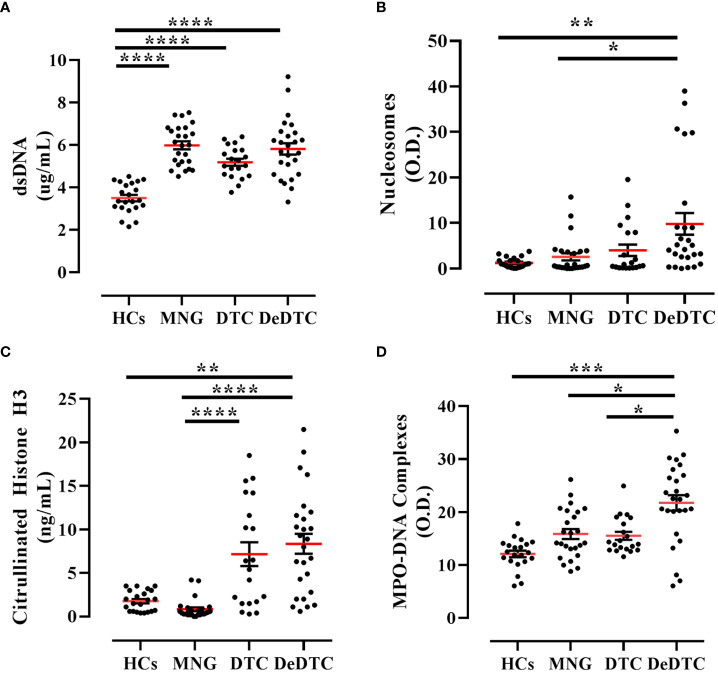
Circulating levels of NET biomarkers in patients with multinodular goiter (MNG), differentiated thyroid cancer (DTC), dedifferentiated thyroid cancer (DeDTC) and healthy controls (HCs). Serum levels of dsDNA **(A)** nucleosomes **(B)**, citrullinated histone H3 **(C)** and MPO-DNA complexes **(D)** were shown. Data are expressed as mean ± SD; One-Way ANOVA and Dunn’s multiple comparison test; *p< 0.05; **p<0.01; ***p<0.005; ****p<0.001.

In a first group of experiments, we measured the serum concentrations of NETs using four different techniques (**Figure 1**). In particular, we evaluated the circulating levels of dsDNA, nucleosomes, CitH3 and MPO-DNA complexes. DeDTC and DTC patients displayed increased levels of dsDNA compared to HCs. MNG patients also displayed increased levels of dsDNA compared to HCs (**Figure 1A**). When we evaluated the serum concentrations of nucleosomes, only DeDTC patients displayed higher levels of NETs compared to both HCs and MNG patients, but they failed to distinguish DeDTC from DTC patients (**Figure 1B**). Both DeDTC and DTC patients displayed higher levels of CitH3 compared to HCs and MNG patients (**Figure 1C**). Finally, we evaluated circulating levels of MPO-DNA complexes in the four groups of donors. Only DeDTC patients displayed higher levels of MPO-DNA complexes compared to HCs, MNG patients and DTC patients (**Figure 1D**). Thus, measurement of MPO-DNA complex levels seems more specific to distinguish DeDTC patients from DTC, MNG or HCs. Serum levels of dsDNA were increased in all three groups of patients. Interestingly, DeDTC patients showed high serum levels of all four NET biomarkers (i.e., dsDNA, nucleosomes, CitH3 and MPO-DNA complexes).

### TC patients display increased circulating levels of neutrophil-derived biomarkers compared to HCs

**Figure 2:**
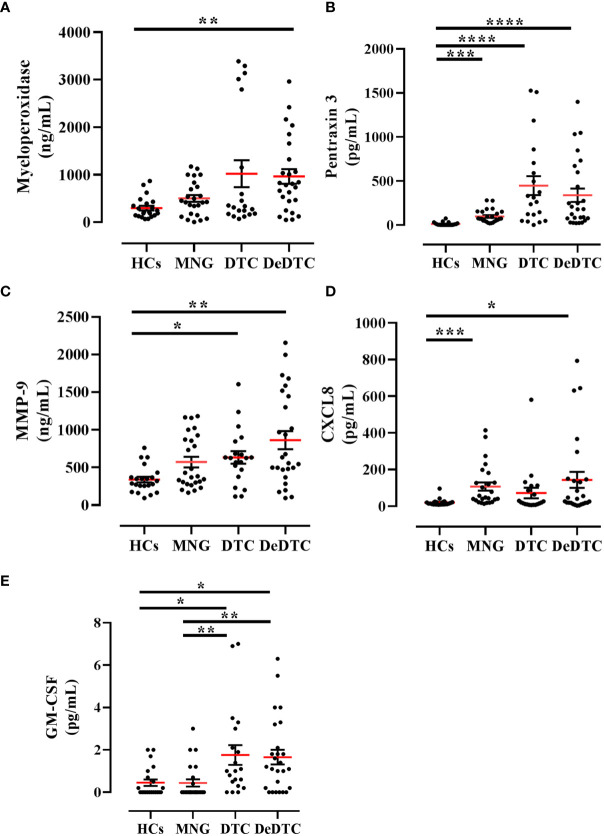
Circulating levels of neutrophil-related biomarkers in patients with multinodular goiter (MNG), differentiated thyroid cancer (DTC) and dedifferentiated thyroid cancer (DeDTC) and healthy controls (HCs). Serum levels of myeloperoxidase **(A)**, pentraxin-3 **(B)**, matrix metalloproteinase-9 (MMP-9) **(C)**, CXCL8 **(D)** and GM-CSF **(E)** were shown. Data are expressed as mean ± SD; One-Way ANOVA and Dunn’s multiple comparison test; *p< 0.05; **p<0.01; ***p<0.005; ****p<0.001.

In the same patient cohort, we measured serum concentrations of several circulating neutrophil-related biomarkers. In particular, we evaluated the circulating levels of three biomarkers of neutrophil granules: MPO derived from azurophilic granules, PTX3 from secondary granules and MMP-9 from tertiary granules (36). We also evaluated circulating levels of CXCL8, the main neutrophil chemoattractant molecule (37) and GM-CSF, one of the main factors promoting neutrophil survival (38, 39). Serum concentrations of MPO, PTX3 and MMP-9 were increased in DeDTC patients compared to HCs (**Figures 2A-C**). PTX3 and MMP-9 levels were also increased in DTC patients compared to HCs (**Figures 2B, C**). Similarly, GM-CSF levels were increased in DTC and DeDTC patients, compared to HCs and MNG subjects (**Figure 2E**). On the other hand, CXCL8 levels were increased in both DeDTC and MNG patients compared to HCs (**Figure 2D**).

### Correlations between circulating levels of NET biomarkers, neutrophil-related mediators and clinic-pathological features in TC patients

**Figure 3:**
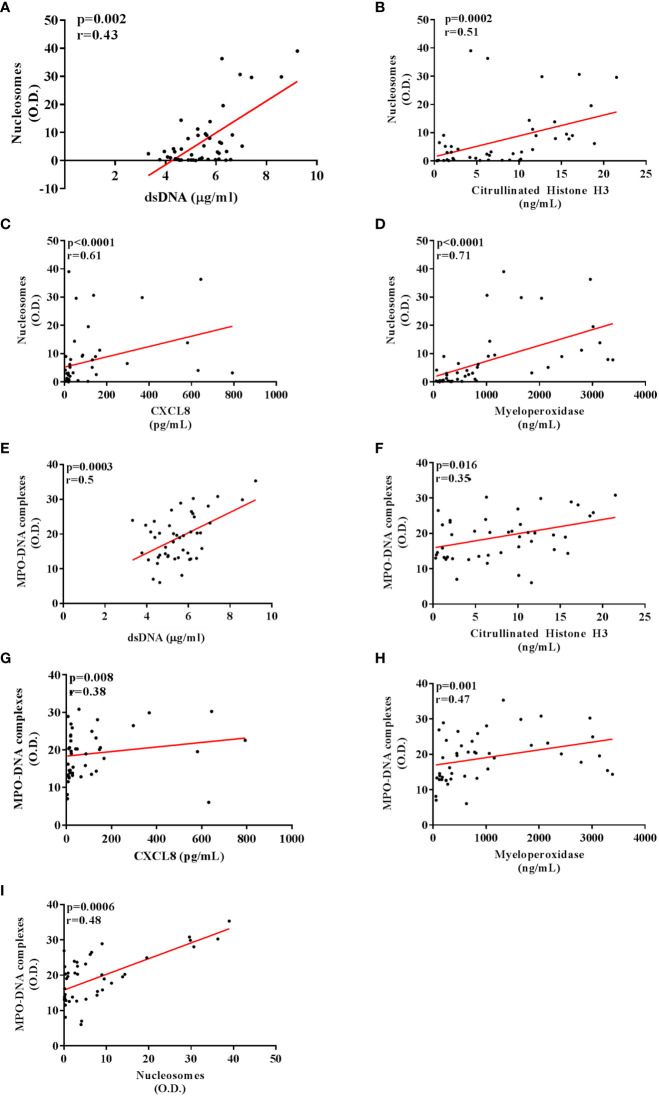
Correlations between circulating levels of NET and neutrophil-related biomarkers in patients with thyroid cancer. Circulating nucleosome levels positively correlated with dsDNA **(A)** and Citrullinated histone H3 **(B)**. A positive correlation was also found between the nucleosome levels and myeloperoxidase **(C)** and CXCL8 **(D)**. Circulating MPO-DNA complex levels positively correlated with dsDNA **(E)**, Citrullinated histone H3 **(F)** and nucleosomes **(I)**. A positive correlation was also found between the MPO-DNA complex levels and myeloperoxidase **(G)** and CXCL8 **(H)**. Pearson or Spearman correlation test. *p* and r were shown in each graph of the figure.

**Figure 4:**
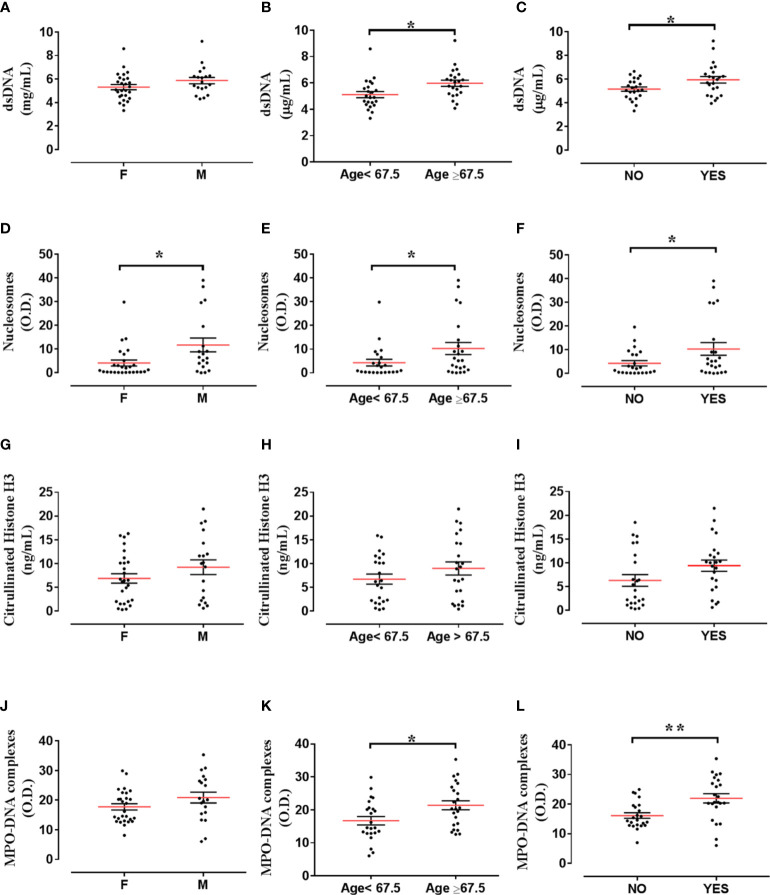
Distribution of NET biomarkers according to patient clinic-pathological features. Distribution of dsDNA levels according to sex [female (F; n=27) versus male (M; n=19)] **(A)**, age [<67,5 (n=23) versus >67,5 years (n=23)] **(B)** and presence of metastasis [without metastasis (NO; n=23) versus with metastasis (YES; n=23)] **(C)**. Distribution of nucleosome levels according to sex [female (F; n=27) versus male (M; n=19)] **(D)**, age [<67,5 (n=23) versus >67,5 years (n=23)] **(E)** and presence of metastasis [without metastasis (NO; n=23) versus with metastasis (YES; n=23)] **(F)**. Distribution of Citrullinated histone H3 levels according to sex [female (F; n=27) versus male (M; n=19)] **(G)**, age [without metastasis (NO; n=23) versus with metastasis (YES; n=23)] **(H)** and presence of metastasis [without metastasis (NO; n=23) versus with metastasis (YES; n=23)] **(I)**. Distribution of MPO-DNA complex levels according to sex [female (F; n=27) versus male (M; n=19)] **(J)**, age [<67,5 (n=23) versus >67,5 years (n=23)] **(K)** and presence of metastasis [without metastasis (NO; n=23) versus with metastasis (YES; n=23)] **(L)**. Unpaired Student’s or Mann-Whitney T test. *p< 0.05; **p<0.01.

We then tested correlations between the circulating levels of NET biomarkers and neutrophil-related mediators in all TC subgroups. Nucleosome levels positively correlated with dsDNA and CitH3 levels (**Figures 3A, B**). Moreover, nucleosome levels correlated with circulating levels of MPO (**Figure 3C**) and CXCL8 (**Figure 3D**). Similarly, circulating levels of MPO-DNA complexes positively correlated with dsDNA (**Figure 3E**), CitH3 (**Figure 3F**), CXCL8 (**Figure 3G**) and MPO (**Figure 3H**). Finally, circulating levels of MPO-DNA complexes also correlated with nucleosomes (**Figure 3I**). We also tested the associations between the circulating levels of NET biomarkers and patient clinic-pathological features. We found that circulating levels of nucleosomes were increased in males compared to females (**Figure 4D**), whereas no correlations were found between circulating levels of dsDNA, CitH3, MPO-DNA and patient sex (**Figures 4A, G, L**). When we distributed patients in two subgroups based on the median value of age, we found that circulating levels of dsDNA, nucleosomes and MPO-DNA complexes were increased in older patients compared to young patients (**Figures 4B, E, M**). No significant differences between the two patient subgroups were found for the circulating levels of CitH3 (**Figure 4H** **).** We also tested the distribution of these four NET biomarkers according to the presence of metastasis. We found that circulating levels of dsDNA, nucleosomes and MPO-DNA complexes were increased in metastatic patients compared to patients without metastasis (**Figures 4C, F, N**). By contrast, CitH3 failed to discriminate between metastatic and non-metastatic patients, even though a trend was observed (**Figure 4I**). These results indicate that nucleosome level was the only biomarker able to discriminate between patient subgroups. Moreover, three out of four NET biomarkers were increased in metastatic patients compared to non-metastatic patients.

## Discussion

In this study, we found for the first time that circulating levels of NET biomarkers and neutrophil-related mediators were significantly increased in TC patients compared to HCs. MPO-DNA complexes and nucleosome levels were selectively increased in DeDTC patients and correlated with dsDNA, CitH3, MPO and CXCL8 levels. Moreover, these two NET biomarkers correlated with each other. Serum levels of MPO-DNA complexes showed differential distribution according to age and presence of metastasis in all TC patient subgroups.

Thyroid cancer is one of the most frequent cancer types worldwide (40). Even though TC survival rates are high in patients with PTC, ATC is a very aggressive subtype with a low survival rate (1). Moreover, DeDTC displays a 3-year survival rate lower than 50% (10). With this in mind, the efforts in looking for biomarkers useful to get early diagnoses of DeDTC and/or predict which tumor will progress are urgently needed.

We recently demonstrated the role of neutrophils (22) and NETs (30) in human TC. No data on the role of circulating NET biomarkers and neutrophil-related mediators in TC patients were available until now. The circulating NET expression can be quantified using different ELISA techniques. From a methodological point of view, a specific and standardized assay of NETs is currently unavailable. For this reason, reports usually refer to NET biomarkers such as circulating levels of free DNA (cfDNA), nucleosomes, circulating Citrullinated Histone H3 (CitH3) and/or NET-related proteins (e.g., NE, MPO). MPO and NE are components and putative biomarkers of NETs, but they are not specific (41). For example, NE and MPO can be released during neutrophil degranulation without NET formation (42). It has been proposed that measuring conjugates like MPO/circulating free DNA (cfDNA) and CitH3 could be more specific for NETs than cfDNA alone (43, 44).

In our cohort of patients, we found that circulating dsDNA levels were increased in DTC as well as in DeDTC patients compared with HCs. Circulating dsDNA levels were also increased in MNG patients suggesting a low specificity for this biomarker in discriminating between benign and malignant TCs. In fact, the quantitative determination of circulating DNA does not necessarily reflect the concentrations of NETs *in vivo*. For instance, DNA complexes may result from other forms of cell death related to cell proliferation (e.g., MNG) or neutrophilic inflammation (45, 46). In our study, we found that CitH3 levels were selectively increased in both DeDTC and DTC patients when compared not only to HCs but also to MNG patients. The latter finding suggests that this biomarker is more specific than dsDNA and can distinguish benign and malignant thyroid tumors. Nucleosomes increased only in DeDTC patients compared to HCs and MNG but failed to distinguish between DeDTC and DTC. Finally, MPO-DNA complex levels were selectively increased in DeDTC patients compared to HCs, MNG and DTC patients. These results suggest that MPO-DNA complex circulating level could be a more specific biomarker of malignancy. Collectively, these findings have translational significance. In fact, a single biomarker is not sufficient in discriminating NET levels in TC patient subgroups.

In this study, we also measured circulating levels of additional neutrophil-related biomarkers. We found that MPO circulating levels were selectively increased in the DeDTC patient subgroup compared to HCs. Circulating levels of both PTX3 and MMP-9 were increased in DTC and DeDTC patients compared to HCs. Similarly, circulating levels of GM-CSF were increased in DTC and DeDTC patients compared to both MNG and HCs. Circulating levels of CXCL8 were increased in DeDTC but also in MNG subjects. The latter result is not surprising since CXCL8 can be produced by a number of cells, including thyrocytes (14).

When we investigated correlations between putative NET biomarkers and neutrophil-related mediators, circulating levels of both nucleosomes and MPO-DNA complexes positively correlated with dsDNA and CitH3, suggesting a good correlation between the four NET biomarkers. In addition, nucleosomes and MPO-DNA complexes also correlated with MPO, in line with the evidence that NETs contain MPO (47). Nucleosomes and MPO-DNA complexes also correlated with serum levels of CXCL8, which could be due to the (active) role of CXCL8 in inducing NET release by human neutrophils (48).

The incidence of PTC has been shown to increase with age (49), and patient age is regarded as an independent risk factor for PTC (50). PTC also presents a poorer prognosis in elderly people (49, 51). Interestingly, PTC is unique among cancers because patient age is part of staging (52–54). When we tested the association between NET biomarkers and patient clinic-pathological characteristics, we found an increase in three out of the four NET biomarkers (i.e., nucleosomes, dsDNA and MPO-DNA complexes) in elderly patients, compared to young patients. A trend was also observed for the distribution of circulating levels of CitH3. These results highlight the possible role of NET biomarkers in assessing the prognosis of DTC patients according to their age. These findings are intriguing because NET formation induced by inflammatory stimuli is impaired in older individuals compared to young adults (55). Collectively, these results indicate that when examining circulating levels of putative NET biomarkers in TC patients and controls, it is important to consider the age of donors.

Among the other clinic-pathological features, nucleosome levels were increased in males compared to females. Moreover, dsDNA, nucleosome and MPO-DNA complex circulating levels were higher in patients with metastatic disease at diagnosis, compared to patients without metastatic disease. A trend towards similar results was also observed for the distribution of CitH3 circulating levels. Collectively, three out of four NET biomarkers were increased in metastatic TC patients, suggesting that NET circulating levels could be related to tumor progression and act as biomarkers of malignancy.

This study suffers from several limitations that should be pointed out. The sample size of the TC patient and healthy control cohorts investigated is limited. Further studies on larger cohorts of healthy donors and patients could underline the significance of circulating levels of NETs in different TCs. Although our results indicate that increased MPO-DNA complex and nucleosomes levels correlated with progressive disease (DeDTC and metastatic patients), at this stage, it would have been inappropriate to define nucleosomes or MPO-DNA complexes as biomarkers of TC progression. Indeed, a larger cohort of patients is needed and survival data related to patient follow-up are needed to get any conclusion.

Noteworthy, different populations of PMNs are prone to produce NETs. For example, low-density neutrophils in patients with systemic lupus erythematosus display enhanced NET formation (56), whereas only a fraction of PMNs from peripheral blood of healthy individuals form NETs, even when stimulated with strong agonists (57). Therefore, an even enhanced NET formation could occur at the tumor site by tumor-associated neutrophils. Although we have not examined the presence of NETs in TC microenvironment, in a previous report we have demonstrated the presence of TC-infiltrating neutrophils which positively correlated with tumor size (22). Future studies should evaluate the presence of intratumoral and peritumoral NETs in TC and whether peripheral blood neutrophils from these patients release more NETs compared to HCs. Moreover, the detection of NET biomarkers is not necessarily an indicator of NETosis but could be related to neutrophil activation or neutrophilic inflammation in TC.

Several immune cells, such as eosinophils (58, 59), basophils (60, 61), mast cells (62–64), and macrophages (65, 66) can form extracellular DNA traps. Therefore, there is the possibility that, in addition to neutrophils, other immune cells involved in TC (10, 12) may contribute to circulating nucleosome levels in these patients.

NETs promote tumor growth (26, 27) and the formation of metastases (29) through different mechanisms. Moreover, it has been shown that NETs partecipate in resistance to radiation therapy (67) and chemotherapy (68) in different tumors. In addition, NETs limit immune response to cancer by impairing contact of immune cytotoxic cells (e.g., CD8^+^ T cells and NK cells) with tumor cells (69).However, recent evidence suggests that NETs, under certain circumstances, can also have an anti-inflammatory role (70, 71). In particular, NETs can promote the resolution of neutrophilic inflammation by degrading cytokines and chemokines (72). Therefore, increasing circulating levels of NET biomarkers and chemokines (i.e., CXCL8) may reflect the complex involvement of neutrophilic inflammation in TC. In conclusion, our study shows for the first time that TC patients display increased circulating levels of NET biomarkers and neutrophil-related mediators. In addition, MPO-DNA complexes and nucleosomes correlated with dsDNA, CitH3, MPO and CXCL8 levels and were increased in elderly patients and patients with metastatic disease at diagnosis. Collectively, these results extend previous findings confirming that neutrophilic inflammation may be involved in thyroid cancer.

## Conflicts of interest

The authors declare that the research was conducted in the absence of any commercial or financial relationships that could be construed as a potential conflict of interest.

## Data availability statement

The raw data supporting the conclusions of this article will be made available by the authors, without undue reservation.

## Ethics statement

The studies involving humans were approved by Ethics Committee for Clinical trials of Pisa University Hospital. The studies were conducted in accordance with the local legislation and institutional requirements. The participants provided their written informed consent to participate in this study.

## Author contributions

Conceptualization, LM and LC. Methodology, LM, LC and AV. Software, LC and RP. Validation, MG, GV and SF. Formal analysis, PF and GV. Investigation, PF, S.RP, SF and LM. Resources, A.A., SP and SF. Data curation, LM, LC, RP. Writing—original draft preparation, LM and MG. Writing—review and editing, MG, GV, GM and AA. Visualization, AA and GM. Supervision, MG, GM. Project administration, MG, GM. Funding acquisition, MG. All authors contributed to the article and approved the submitted version.
