# Supplementary material for: Neutrophil extracellular traps and neutrophil-related mediators in human thyroid cancer
Source: Front Immunol. 2023 Aug 29;14:1167404. doi: 10.3389/fimmu.2023.1167404 (PMC10495767; doi:10.3389/fimmu.2023.1167404)
Supplement: Supplementary file 1 [file Table_1.docx]

**Supplementary Table 1**. Demographic and clinic-pathological features of TC patients.

|  | ***N*** | **%** |
| --- | --- | --- |
| **Age** |  |  |
| Median, years**^#^**  Range | 67.5  37-97 |  |
| **Gender** |  |  |
| Male | 19 | 41.3 |
| Female | 27 | 58.7 |
| **Cancer Type** |  |  |
| Differentiated Thyroid Cancer (DTC) | 20 | 43.5 |
| Stage I | 17 | 85.0 |
| Stage II | 3 | 15.0 |
| DeDifferentiated Thyroid Cancer (DeDTC) | 26 | 56.5 |
| Stage I | 8 | 30.8 |
| Stage II | 6 | 23.1 |
| Stage III | 3 | 11.5 |
| Stage IV | 9 | 34.6 |

**N** number; **#**Age entered as continuous variable
